# Efficacy and safety profile of a novel technique, ThuLEP (Thulium laser enucleation of the prostate) for the treatment of benign prostate hypertrophy. Our experience on 148 patients

**DOI:** 10.1186/1471-2482-12-S1-S21

**Published:** 2012-11-15

**Authors:** Fabrizio Iacono, Domenico Prezioso, Giovanni Di Lauro, Giuseppe Romeo, Antonio Ruffo, Ester Illiano, Bruno Amato

**Affiliations:** 1 Department of Urology, School of Medicine, University “Federico II” of Naples, Via pasini, 5 – 80131 – Naples, Italy; 2 Department of Urology, Hospital S.Maria delle Grazie of Pozzuoli (Na), Via Domiziana – 80078 – Pozzuoli (Na), Italy; 3 Department of General, Geriatric, Oncological Surgery and Advanced Technologies, University “Federico II” of Naples. Via Pasini, 5 – 80131 – Naples, Italy

## Abstract

**Background:**

Over the past years laser technology has played a predominant role in prostate surgery, for the treatment of benign prostate hypertrophy (BPH). Various laser devices have been introduced in clinical practice, showing good results in terms of complications and urodynamic outcomes efficacy compared with TURP and Open Prostatectomy.

In this study we describe the efficacy and the safety profile of a novel laser technique, ThuLEP (Thulium Laser Enucleation of Prostate) that permits a complete anatomical endoscopic enucleation of prostatic adenoma independently to prostate size.

**Methods:**

148 patients with a mean age of 68.2 years were enrolled between September 2009 and March 2012 (36 months), and treated for BPH with ThuLEP. Every patient was evaluated at base line according to: Digital Rectal Examination (DRE), prostate volume, Post-Voided volume (PVR), International Prostate Symptoms Score (I-PSS), International Index of Erectile Function-5 (IIEF-5), Quality of Life (QoL), PSA values, urine analysis and urine culture, uroflowmetry. The same evaluation was conducted after a 12 month follow-up. ThuLEP was performed by 2 expert surgeons.

**Results:**

Our data showed a better post-operative outcome in terms of catheter removal, blood loss, TURP syndrome, clot retention and residual tissue compared to large series of TURP and OP. Only 1.3% of patients had bladder wall injury during morcellation. I-PSS, Qmax, Prostate Volume, QoL and PVR showed a highly significant improvement at 12 month follow-up in comparison to preoperative assessment.

**Conclusion:**

ThuLEP represent an innovative option in patients with BPH. It is a size independent surgical endoscopic technique and it can be considered the real alternative, at this time, to TURP and even more to Open Prostatectomy for large prostate, with a complete removal of adenoma and with a low complication rate.

## Background

Over the past decade laser endoscopic surgery has played a prominent role as an alternative to TURP and open prostatectomy (OP) for the treatment of benign prostatic enlargement (BPE) and obstruction (BPO) [1,2].

Various laser devices have been introduced in clinical practice during these years, and four groups of laser systems are currently used: KTP (kalium titanyl phosphate, KTP:Nd:YAG [SHG]) and LBO (lithium borat, LBO:Nd:YAG [SHG]); Diode lasers (various); Holmium yttrium-aluminum-garnet laser (Ho:YAG) and Thulium YAG (Tm-YAG) the others have been superseded [3,4].

All of these transurethral laser operations need a physiologic sodium solution 0.9% for irrigation, and this eliminates the risk of hypotonic hypervolaemic TURP syndrome, that has been reported in large TURP series [5]. Furthermore, they offer the advantage of decreased bleeding complications and the possibility to treat patients with bleeding disorders or anti-coagulative treatment [6].

The aim of this study is to present and to prove the efficacy and safety profile of a novel technique , ThuLEP (Thulium laser enucleation of the prostate), that permits complete transurethral removal of the transition zone (enucleation) with the support of the Thulium laser to combine complete anatomical enucleation with maximum urodynamic outcome efficacy and with minimal side effects.

The first Thulium laser was used in clinical practice in 2005 after introduction of the Holmium laser, and since then has become the most innovative laser technology in urology [7]. The Thulium laser offers the advantage to work in continuous wave (cw) mode at a wavelength of two microns. The Thulium laser offers complete absorption of laser energy in water. Tm-TAG is suitable for many transurethral prostate surgical techniques such prostate vaporization (ThuVAP), bladder neck incision [8], Vaporesection (ThuVARP) [9,10]. It is also indicated for Vapoenucleation of the prostate [6] that permits a complete removal of the prostatic adenoma using a blunt mechanical enucleation of the tissue. For some aspects it is like the “index finger" in open surgery for large prostate but with the advantages of laser energy to reduce bleeding and achieve safe hemostasis using the Thulium laser.

## Methods

We evaluated 148 patients with a mean age of 68.2 ± 5.03 yrs. (47yrs.-85yrs.) treated for BPH with symptomatic LUTS, using the ThuLEP technique between September 2009 and March 2012 (36 months). Inclusion criteria were: prostate volume 75 cc or greater; urinary flow rate (Qmax) not greater than 15 ml/sec; an IPSS > 7. We excluded patients with prostate cancer, neurogenic bladder dysfunction diagnosed by urodynamic evaluation and previous lower urinary tract surgery.

We evaluated all patients according to: Digital Rectal Examination (DRE), prostate volume by TRUS, Post-Voided Volume (PVR) by abdominal ultrasonography, International Prostate Symptoms Score (I-PSS) by self- administered questionnaire, International Index of Erectile Function-5 (IIEF-5), quality of life (QoL) by self-administrated questionnaire, PSA assay, urine Analysis and urine Culture. Every patient has performed uroflowmetry, except those in urinary retention, to determinate Qmax. Patients with suspicious PSA or with suspicious DRE, underwent a randomized 12-core needle ultrasonography-guided trans-rectal prostate biopsy.

ThuLEP was performed by 2 surgeons with experience with more than 70 procedures and with previous long experience in endourology for the BPH endoscopic surgery (TURP – HoLEP –ThuVEP). Most of the patients had spinal anesthesia, except those with failure of regional anesthesia and failed spinal anesthesia, in these patients general anesthesia was used.

Here below is described the surgical technique.

### Equipment

We used a 26 Fr continuous-flow resectoscope (Karl Storz GmbH, Tuttlingen, Germany) with a separate working channel with a 12° lens system visualized the lower urinary tract. A camera (Karl Storz GmbH, Tuttlingen, Germany) was connected to the lens for image enlargement and High Definition (HD) video to document the procedure. A 2-lm cw-Thulium:YAG laser (RevolixTM 120 W surgical laser, LISA laser products, Katlenburg, Germany)was used with a re-usable 550 lm laser fibre (RigiFibTM, LISA laser products, Katlenburg, Germany) with a blunt spike, to apply laser energy. The energy was released at 120 W for incision of the lateral margins of the median lobe and at the verum montanum and 40 W for coagulation of capsule perforating vessels during blunt enucleation of the prostatic adenoma. Physiological saline solution at 0,9% was used for irrigation during the entire procedure.

A mechanical tissue morcellator (Piranha, Richard Wolf GmbH, Knittlingen, Germany) was used for fragmentation in small slices of the prostatic adenoma. The morcellator worked into the bladder lumen that was distended during the procedure to avoid bladder wall injuries.

The morcellator unit consists of a mechanical hand-piece, control unit, rotating blades with a diameter of 5 mm, suction pump, activated by a foot pedal. The morcellator has to be inserted into the bladder through a nephroscope that is connected to the outer sheath of the resectoscope through an adapter (Karl Storz GmbH, Tuttlingen, Germany). A double inflow allowed the bladder distention.

### Surgical technique

The patient is placed in the lithotomic position. Sterile draping of the patients is prepared while sterile gel is put in the urethra. The resectoscope is inserted, under vision, into the bladder. It is recommendable to perform a cystoscopy in the way to exclude eventually bladder pathologies and to have a look at the ureteral orifices. Finally the resectoscope is pulled back into the prostatic urethra and a detailed evaluation is made of the bladder neck, the extent of lobar protrusion, position of verum montanum and the borders of external urethral sphincter.

The enucleation starts with removal of the prostatic median lobe. An inverted U-incision at the level of the verum montanum is placed, to delimitate the distal board of resection. Bilateral bladder neck incisions close to the lateral margins of the prostatic median lobe are made at the 5 and 7 o’clock positions. These incisions are extended until the distal third of the verum montanum, board the entire median lobe. Finally a deeper incision can be made so the surgical capsule can be visible. It is a white layer with superficial small vessels. At this time, it is possible to start the blunt retrograde enucleation of the prostatic median lobe. The enucleation technique is performed with the resectoscope that is used like a retractor, that is pushed and bluntly shifted towards the 12 o'clock direction the median lobe. This action has to be conducted under the edge of the median lobe that is softly separated from the surgical capsule. The surgical capsule is used as a natural cleavage plane, as has been done with an index finger in the open prostatectomy. During blunt disconnection of the adenoma, laser coagulation of perforating vessels of the surgical capsule is necessary and needs to be continued until the bladder neck is gained. When complete disconnection of the median lobe from the surgical capsule is reached, the adenoma can be pushed into the bladder lumen.

The lateral lobes are removed separately, beginning with the smaller one if the adenoma is asymmetric, we begin conventionally from the left lobe for symmetric adenoma. The first incision is made at the distal margin of the adenoma at the 12 o’clock position. From the U-inverted incision, two superficial incisions towards the 4 o'clock (left lobe) and 8 o'clock (right lobe) positions are carried out. The apical board of the lateral lobes is then incised between the incisions at 12 o'clock position and the incision at 4 o'clock or 8 o'clock. At this time the bluntly shifted process can be done in the same way we described for the median lobe, but in this case it is necessary to pull the lateral left lobe towards the 2 o'clock position and the lateral right lobe at 10 o'clock into the bladder lumen. The surgical capsule can be identified by visualizing the white layer and small perforating vessels. It is necessary to coagulate these small vessels and use a 40 W energy until the bladder neck is reached. Again, after complete release from the surgical capsule, the lateral lobe is pushed into the bladder. The same procedure is then identically repeated on the other side.

When the entire adenoma has been pushed into the bladder lumen the resectoscope is replaced by a nephroscope adapted for the morcellator. The suction pump attracts the pieces of the adenoma and the blades fragment them. This procedure is done with continuous irrigation and a fully distended bladder to avoid any bladder wall injuries. The procedure ends with a 22 Fr catheter.

The catheter was removed after 48 hours from the surgery. Patients were discharged home after a successful voiding trial after the catheter removal. All complications were recorded and blood loss was estimated by hemoglobin blood evaluation 24 hours after surgery. Enucleated tissue was histopathologically evaluated in all cases.

Post-surgery follow-up was conducted after 12 months from surgery and each patient was evaluated for TRUS, PSA value, Qmax, PVR, IPSS, IIEF-5 and QoL. All patients were asked about any complication encountered during the previous 12 months.

Statistical analysis was performed by the program Statistical Package for Social Sciences for Windows, version 11.5.1 (SPSS Inc., Chicago, IL, USA).

We expressed patient data according to: mean +/- SD or as median with interquartile range. We calculated data paired between pre and post-operative using t-test with p<0,05 considered statistically significant.

## Results and discussion

In Table 1 there is the list of baseline and follow-up characteristics of the patients. Thirty-nine patients (26.3%) had urinary retention before surgery and were not able to void without catheter. 105 patients (70.9%) had a gland volume > 90 gr. Table 2 lists perioperative data. The ThuLEP procedure was successfully completed in all patients. None of the patients had ureteric orifice injury, TURP syndrome, clot retention or incomplete morcellation. In 2 patients (1.3%) a bladder wall injury during morcellation occurs. 4 patients (2.7%) required early recatheterization after surgery and cystoscopy showed residual tissue at the apex of prostate fossa. 4 patients (2.7%) needed early postoperative blood transfusion due to persistent hematuria with continuous bladder irrigation and prolonged catheterization. 10 patients (6.7%) had postoperative irritative symptoms with temporary urge incontinence, by the way none of them had these symptoms at 12 months follow-up. UTI occurred in 19 patients (12.8%) everyone of them received adequate antibiotic therapy. 2 patients developed urethral stricture during follow-up, they needed surgery with cold incision of the stricture part.

**Table 1:** 

	Preoperative Mean ± SD (Range)	Postoperative Mean ± SD (Range)
No. of patients	148	122

Age (years)	68, 2 ± 5,03 (47 – 85)	

PSA (ng/ml)	9,53 ± 8,32 (1,5- 40,2)	0,93 ± 0,67 (0,12 – 3,56)

Prostate Volume (ml)	108,08 ± 24,23 (75 – 210)	13,76 ± 9,47 (4 – 43)

IPSS	21,10 ± 7,12 (8 – 35)	3,90 ± 2,42 (0 – 14)

QoL	4,38 ± 1, 32 (1 – 6)	0,94 ± 0,67 (0 – 4)

Qmax (ml/sec)	8, 23 ± 3,65 (1,3 – 15)	28,67 ± 10,67 (15 – 56)

PVR (ml)	146,12 ± 132,32 (40 – 600)	12,89 ± 20,87 (1 – 128)

IIEF-5 score	19,3 ± 8,23 (6- 30)	20,3 ± 8,16 (6 – 30)

		*p< 0*,*05*

**Table 2:** 

	Mean ± SD (Range)
No. of patients	148

Operation duration (min)	70,03 ± 25,87 (40 – 150)

Enucleation duration (min)	50,34 ± 28,76 (15 – 110)

Morcellation duration (min)	18,23 ± 13,34 (8 – 60)

Enucleation efficiency (weight/laser duration) (g/min)	2,34 ± 0,87 (0,97 – 5,34)

Morcellation efficiency (weight/morcellation duration) (g/min)	5,23 ± 1,04 (1,32 – 12,34)

Haemoglobin decrease (g/dl)	1,27 ± 0,88 (0,53 – 4,55)

Catheter time (days)	2,04 ± 0,45 (1 – 7)

Hospitalization (days)	2,15 ± 0,39 (2 – 5)

Histopathological examination of the enucleated tissue showed an incidental adenocarcinoma of the prostate in 8 patients (5.4%) and a benign prostate hyperplasia in 140 patients (94.5%). Four patients died during follow-up, while 14 did not come to the follow-up evaluation, they were phoned and asked if any complication occurred during the follow-up. Patients with adenocarcinoma of the prostate were excluded from the 12 month follow-up control and from further analysis they were treated separately according to their oncological situation. Therefore of the 148 eligible patients, only 122 of them were available at 12 months follow-up.

I-PSS, Qmax, prostate volume, QoL and PVR showed a highly significant improvement at 12 months follow-up in comparison to preoperative assessment.

BPO with all correlate symptoms (LUTS) is one of the most frequent pathologies in urological daily practice. Transurethral resection of the prostate (TURP) in all its various forms is the predominant procedure performed worldwide for small adenomas. Open simple prostatectomy is the treatment option for large prostate. However, both methods are associated with relevant morbidity. A review of 9,538 patients, compared surgical outcome between TURP and OP showed an overall complication rate(blood transfusion, epididymitis, bladder neck contraction, uretral stricture and postoperative erectile dysfunction) of 15% for TURP and 21% for OP [95% CI 7.0-42.7] [11]. Another large series of 10,654 patients showed that TURP has an overall morbidity rate of 11.1% and an overall mortality rate of 0.1%. The most frequent complications were in order: failure to void (5.8%), surgical revision due to bleeding (5.6%), requiring blood transfusion (2.9%), TUR syndrome (1.4%) [12].

On the other hand OP showed in a recent series a complication rate of 15%, with a rate of severe bleeding of 12% with a transfusion rate approximately of 8.2%; this series showed a catheterization time of 7 days [13]. One series reports a transfusion rate up to 26.5% for OP [14]. Therefore new techniques have been proposed during the last 5 years, with the improvement of surgical technology. Laser-based treatment such as HoLEP showed in randomized controlled trials, a significant decreased in morbidity, with a good release of obstructive symptoms, and a totally size-independent method for the treatment of BPO [15,16].

The aim of our study is to evaluate the efficacy of a new technique (ThuLEP) developed for the first time in 2009 by Imkamp et al. [17]. However no data are yet dealt with this kind of transurethral approach comparing it to other laser techniques or compared with “more standard” techniques. Our data show a good safe profile for ThuLEP, the most common complication rates were in order: UTI (12.8%), irritating symptoms (6.7%), re-catheterization (2.7%). Our data show a transfusion rate of 2.7% and a re-catheterization rate of 2.7%. Bladder wall injury was 1.3%.

It should be stressed that this procedure is independent from the prostate size. We treated patients from 75 to 210 gr. of prostate, despite TURP and OP. Some authors have showed an increased risk of complication and increased risk of morbidity when large volume prostates are treated [18]. ThuLEP was introduced to overcome these problems with the same or even better results in term of overall complication rate and overall survival rate.

We believe that ThuLEP will be, in the future, the alternative of TURP and of OP to treat BPO because it offers the advantages of endoscopic, minimally invasive surgical intervention, with the advantages of anatomical blunt dissection of the adenoma like the index finger in the open prostatectomy, with a small complication rate. Laser energy is used only to describe the correct border of dissection at the prostate apex, at the bladder neck and at the prostate lobes. Furthemore Thulium YAG laser offers a maximum haemostasis for three reasons. The first reason is that ThuYAG energy is delivered in a continuous-wave mode, which can provide excellent coagulation [19].

The second reason has been explained by Bach et al. [20] that showed a better bleeding control using 120-W Tm:YAG device despite 70-W Tm:YAG device. The third reason is that in ThuLEP the adenoma is a blunt dissect with the resectoscope. This allows the surgeon to see and to coagulate vessels that come out from the prostate surgical capsule during the dissection, with maximum control of the haemostasis [6,17].

Furthermore with ThuLEP it is expected that no prostatic tissue of the adenoma is left behind. This should imply a better outcome in terms of uroflowmetry, postvoid residual urine, IPSS, and re-treatment. Capsule perforation is almost impossible because the surgical capsule is always visible, and this decreases the possibility of erectile dysfunction due to damage of the neurovascular bandels due to a pseudocapsule perforation at the level of the lateral lobes.

## Conclusions

ThuLEP represent a safe, effective surgical option in patients with symptomatic BPH. It can be considered, at this time, the real alternative to TURP and even more to OP for large prostate. Our data showed that this technique is size-independent, with complete removal of the adenoma, reduction in TRUS volume and PSA value in this way it can be compared to open prostatectomy. It is also safe, with a low complication rate and it can be used in patients under anticoagulant/antiplatelet therapy.

In expert hands morcellation does not seem to improve the complication rate.

Further studies are needed for this technique to enter into daily surgical practice.

## List of abbreviations

BPE: Benign-Prostate-Enlargement; BPH: Benign-Prostate-Hypertrophy; BPO: Benign-Prostate-Obstruction; DRE: Digital-Rectal-Examination; HoLEP: Holmium-Laser-Enucleation-of-Prostate; Ho:YAG: Holmium-Yttrium-Aluminium-Garnet; IIEF-5: International-Index-of-Erectile-Function; I-PSS: International-Prostatic-Symptoms-Score; LUTS: Lower-Urinary-Tract-Symptoms; OP: Open-Prostatectomy; PSA: Prostatic-Specific-Antigen; PVR: Post-Void-Residue-urine-volume; Qmax: Maximum-Flow-Rate; QoL: Quality-of-Life; ThuLEP: Thulium-Laser-Enucleation-of-Prostate; ThuVAP: Thulium-Laser-Vaporization-of-Prostate; ThuVARP: Thulium-laser-Vapo-Resection-of-Prostate; ThuVEP: Thulium-laser-Vapo-Enucleation-of-Prostate; Tm:YAG: Thulimu-Yttrium-Aluminium-Garnet; TRUS: Trans-Rectal-Ultrasonography; TURP: Trans-Urethral-Resection-of-Prostate.

## Competing interests

The authors declare that they have no competing interests.

## Authors’ contributions

FI: conception and design, critical revision, interpretation of data, given final approval of the version to be published; DP, GdL: conception and design, critical revision, given final approval of the version to be published; GR, AR, EI: acquisition of data, drafting the manuscript, given final approval of the version to be published; BA: critical revision, interpretation of data, given final approval of the version to be published.
